# Design and Research of an Underactuated Manipulator Based on the Metamorphic Mechanism

**DOI:** 10.3390/s22134766

**Published:** 2022-06-24

**Authors:** Lele Sun, Haiou Zhang, Hang Lin, Wujiu Pan

**Affiliations:** 1 School of Mechanical Science and Engineering, Huazhong University of Science and Technology, Wuhan 430074, China; d201880273@hust.edu.cn (L.S.); hanglin@hust.edu.cn (H.L.); 2 School of Mechatronics Engineering, Shenyang Aerospace University, Shenyang 110136, China; panspace@sina.cn

**Keywords:** kinematic analysis, manipulator, metamorphic mechanism, underactuated finger mechanism

## Abstract

Robot hands play an important role in the interaction between robots and the environment, and the precision and complexity of their tasks in work production are becoming higher and higher. However, because the traditional manipulator has too many driving components, complex control, and a lack of versatility, it is difficult to solve the contradiction between the degrees of freedom, weight, flexibility, and grasping ability. The existing manipulator has difficulty meeting the diversified requirements of a simple structure, a large grasping force, and the ability to automatically adapt to shape when grasping an object. To solve this problem, we designed a kind of underactuated manipulator with a simple structure and strong generality based on the metamorphic mechanism principle. First, the mechanism of the manipulator was designed on the basis of the metamorphic mechanism principle, and a kinematics analysis was carried out. Then, the genetic algorithm was used to optimize the size parameters of the manipulator finger structure. Finally, for different shapes of objects, the design of the control circuit binding force feedback control was carried out with a grasping experiment. The experimental results show that the manipulator has simple control and can grasp objects of different sizes, positions, and shapes.

## 1. Introduction

With the wide application of robots in industrial production and human life, the requirements for their grasping ability are becoming higher and higher. Robot hands can imitate the basic functions of the human hand; they are widely used in the aerospace [1,2] and industrial sectors [3]. Robot hands are usually loaded at the end of the mechanical arm, also known as the end-effector [4]. In the past 30 years, the development of robot hands has received great attention from researchers [5]. Many scholars have made outstanding achievements in the field of robot hands. As one of the most important components of a robot system, the end manipulator has important research significance. According to the relationship between the number of actuators and the number of degrees of freedom, the robot hand is divided into the redundant hand, the fully actuated hand, and the underactuated hand. Among them, redundancy and full drive design methods are widely used in the field of dexterous hands. Their characteristic is that they can accurately control the position and posture of the fingers by controlling the movement of each finger joint. 

In recent years, researchers have developed many dexterous hands, including the Utah/MIT hand [6], TUAT/Karlsruhe hand [7], and BCL-13 [8]. These dexterous hands can complete complex grasping tasks. However, because each joint degree of freedom is driven by at least one actuator, and a complex control and sensing system is usually used to manage the whole equipment, the redundant and full drive manipulators are too heavy and expensive. These shortcomings limit the practicability of these manipulators. Therefore, it is necessary to design a manipulator with relatively simple control and low weight on the premise of ensuring various complex grasping tasks [9]. This has led to the development of underactuated fingers, for example, SDM [10], PASA-GB hand [11], and SARAH [12,13,14,15].

At present, underactuated technology is one of the main research directions in the development of anthropomorphic robots [16]. The underactuated manipulator adopts the driving mode of the combination of active motor and passive components. The number of actuators required by the fingers of this manipulator is less than the number of degrees of freedom they have. Instead, the spring or coupling mechanism is used to realize the underactuated grasping function [17]. This design enables the underactuated hand to carry out multi-level grasping, and the preloaded spring is used to passively control the movement of the hand until the underactuated hand contacts the grasped object. It can adaptively grasp objects of different shapes and sizes, which solves the problem of difficult control [18].

Through the analysis of the above literature, redundant drive and full drive manipulator usually set a drive and sensor for each degree of freedom, resulting in too many drive components and complex control, reducing the flexibility and controllability of the manipulator, causing a lack of generality, and increasing the development cost. How to solve the contradiction between the manipulator’s degrees of freedom, driving mode, flexibility, grasping ability, and reliability has become a key problem in the research and development of new manipulators. To solve this problem, this paper draws on the idea of active and passive composite drive [19], uses the driving mode combining active motor and passive components, and designs an underactuated manipulator based on the metamorphic principle. The manipulator adopts the active drive of a single motor and the passive drive of spring to realize the adaptive grasping of objects of different shapes. It has the advantages of compact structure, simple control, and strong adaptability. Compared with the existing literature, this paper mainly includes the following four innovations: (1) underdrive with only one drive; (2) reasonable structure—the contact force model is established and the structural parameters of the finger are optimized so that under the limited driving force, the force of the finger knuckles is as uniform as possible, and the grasping force of the object is large enough; (3) adaptivity—the underactuated manipulator has finger branch azimuth adaptive mechanism by which two fingers can rotate around their axis of rotation 60° and carry out the adaptive grasping of objects of different shapes; (4) serialization—the length of each finger joint can be determined by the size of the object to grab, and in practical application, the manipulator can be developed into a series of products.

This paper consists of four parts. After the introduction, Section 2 establishes the structural model of the underactuated manipulator, carries out a kinematic analysis, and analyzes the different isomorphic states of the manipulator’s fingers in the process of grasping objects. To help the design and optimization of underactuated fingers, a static model of fingers was established, and the contact force was analyzed. The finger structure parameters are optimized to generate enough grasping force under the action of the limited driving force to achieve the reliable grasping of objects. In addition, to make the contact force between the three fingers and the object more uniform, this study used the program of a genetic algorithm to optimize the parameters of finger structure. In Section 3, the corresponding control system is designed, and the physical model of the underactuated manipulator is made. The grasping test is carried out on objects of different sizes and different materials to evaluate the underactuated manipulator with good adaptability. Section 4 presents the conclusion.

## 2. Proposed Method

**Figure 1:**
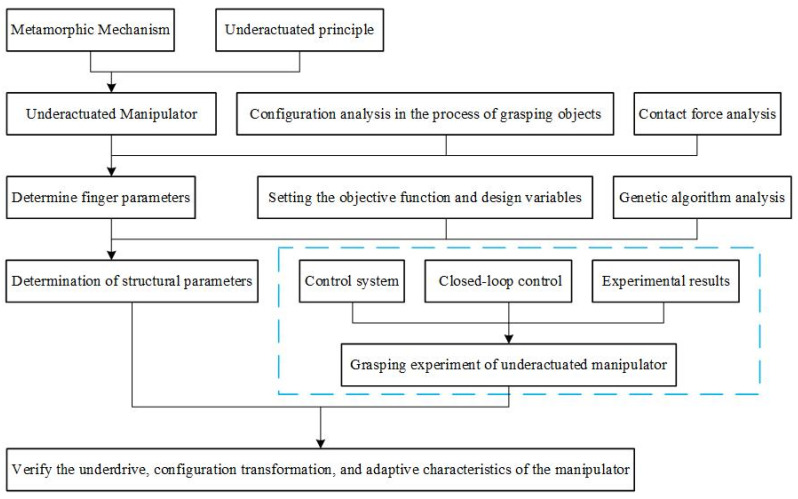
Flow diagram of the underactuated manipulator design method.

The flow diagram of the underactuated manipulator design method based on the metamorphic mechanism proposed in this paper is shown in Figure 1. We first describe the metamorphic mechanism and the underactuated principle, which was used to establish the underactuated manipulator model. Then, we analyzed the configuration of fingers in different stages during the grasping process of the manipulator. To provide help for the design and optimization of the underactuated finger, the contact force of the finger was analyzed according to the static model of the finger and the principle of virtual work. Furthermore, we set up the objective function and design variables and used the genetic algorithm to optimize the finger structure parameters of the underactuated manipulator. In this way, the underactuated manipulator could grasp the stability and the finger knuckle’s contact force as uniformly as possible. The underactuated manipulator model, kinematics analysis, contact force analysis, and finger structural parameter optimization mentioned in the proposed method are detailed as follows.

### 2.1. Structure and Working Principle of the Underactuated Manipulator

In the development process of robot dexterous hands, most mechanical hands are rigid bodies and rely on the movement of fingers to grasp, which limits the range of movement and dexterity of fingers. To improve the flexibility of the manipulator, only the method of increasing the number of fingers has been adopted in the past. To break through the limitation of the fixed palm of the traditional manipulator, this study designed an underactuated manipulator based on the principle of the mutation cell, which is driven by a motor and controlled by the manipulator.

In the cycle with multiple working stages, the multi-degree-of-freedom motion chain with a closed loop presents different topological structure forms, and the mechanism with different functions by combining its frame and original moving parts is called the metamorphic mechanism.

When the number of actuators of a mechanism is less than the number of degrees of freedom of the mechanism, the mechanism is underactuated.

**Figure 2:**
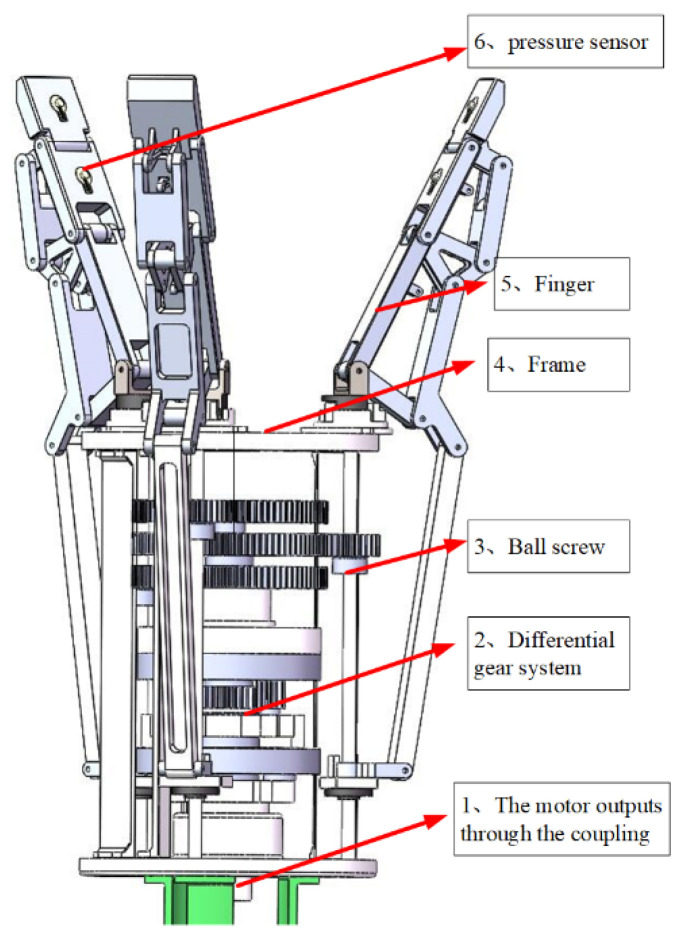
General assembly drawing of the manipulator.

The overall structure of the underdrive hand is shown in Figure 2. The underdrive manipulator is composed of a driving motor, a transmission mechanism (ball screw and differential gear system), a frame, and three underdrive fingers. The manipulator uses a motor to drive three fingers to complete the grasp of the object, with 9 degrees of freedom.

**Figure 3:**
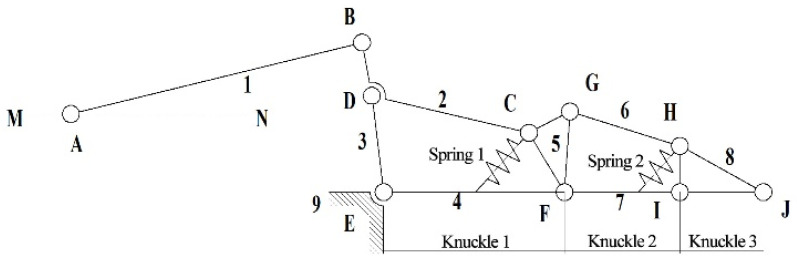
Finger mechanism of the underactuated manipulator.

The underactuated finger, as shown in Figure 3, consists of two tension springs and nine connecting rods. Tension spring 1 is located between rods 4 and 5. Tension spring 2 is located between rods 7 and 8. Rods 4, 7, and 8 represent the first, second, and third knuckles, respectively, and rod 9 is the frame. The revolute joints are points B, C, D, E, F, G, H, and I. The linear drive is located at point A, and drive point A moves along the dotted line MN.

When the finger mechanism of the underactuated manipulator grasps the object, the drive moves along the direction of MN. Connecting rod 1 rotates through joint A, connecting rod 2 moves through joint B, connecting rod 3 moves through joint D, and connecting rod 4 (first knuckle) moves through joint E. Link two passes through joint C, and link four passes through joint F to move link five. The G joint drives the motion of link 6, and the F joint drives the motion of link 7 (second knuckle). Connecting rod 6 passes through the H joint while connecting rod 7 drives connecting rod 8 (third knuckle) through the I joint until the finger is in contact with the grabbed object and the envelope grasp is completed.

### 2.2. Kinematic Analysis of Grasping Objects with Fingers

To analyze the movement of the finger mechanism based on the metamorphic mechanism in the grasping process, finger states in different grasping stages are divided into the first configuration, the second configuration, and the third configuration.

First configuration: the finger mechanism is initially in a free state, that is, all knuckles have no contact with the object being grabbed.

Second configuration: the first knuckle of the finger mechanism is in contact with the object; the first knuckle is fixed, and the second and third knuckles rotate and are always in the same plane.

In the third configuration, the first and second knuckles of the finger mechanism are in contact with the object, and the third knuckle rotates.

#### 2.2.1. Kinematics Modeling in the First Configuration

**Figure 4:**
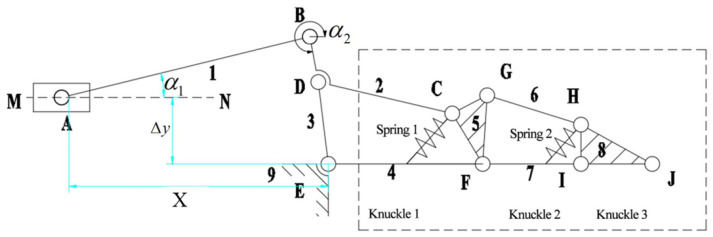
Schematic diagram of the equivalent mechanism of the first configuration.

The finger mechanism is active at the beginning and can be simplified as an equivalent crank slider mechanism ABE, as shown in Figure 4. $l_{i}$ represents the rod length, i=1,2…,9. $\alpha_{i}$ represents the angle variable between the forward direction of the X-axis and each bar.

The original moving part is the slide block, and its moving speed is Δx/s. Then, the motion rule of the slide block is $X=x_{0}-(\Delta x)t$, where $x_{0}$ is the initial value of $l_{AE}$. The crank slider mechanism offset size is Δy.

According to the geometrical relation between ${\vec{l}}_{AB}$ and ${\vec{l}}_{BE}$, the vector equation can be obtained: ${\vec{l}}_{AB}+{\vec{l}}_{BE}={\vec{l}}_{AE}$.

It is derived from Euler’s equations:

(1) $$\left\{ \begin{array}{l} l_{AB}\cos\alpha_{1}+l_{BE}\cos\alpha_{2}=X\\ l_{AB}\sin\alpha_{1}+\Delta y+l_{BE}\sin\alpha_{2}=0 \end{array}\right.$$

Subtracting $\alpha_{2}$ from (1) gives:

(2) $$l_{BE}^{2}=X^{2}+l_{AB}^{2}-2l_{AB}X\cos\alpha_{1}+2l_{AB}\Delta y\sin\alpha_{1}$$

It can be solved from (2) that:

(3) $$\alpha_{1}=\arcsin((bc\pm a\sqrt{a^{2}+b^{2}-c^{2}})/(a^{2}+b^{2})).$$

It should be taken in the crank slider mechanism:

(4) $$\alpha_{1}=\arcsin((bc+a\sqrt{a^{2}+b^{2}-c^{2}})/(a^{2}+b^{2})).$$

Substituting into (1) obtains:

(5) $$\alpha_{2}=\arcsin((-l_{AB}\sin\alpha_{1}-\Delta y)/l_{BE}.$$

In the equation above,

$$a=2l_{AB}X,\;b=2l_{AB}\Delta y,\;c=l_{BE}^{2}-l_{AB}^{2}-X^{2}.$$

#### 2.2.2. Kinematics Modeling in the Second Configuration

**Figure 5:**
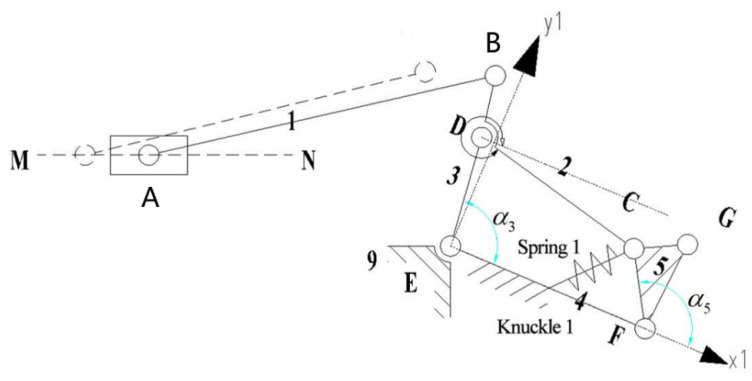
Schematic diagram of the equivalent mechanism of the second configuration.

When the first knuckle is in contact with the captured object, as shown in Figure 5.the finger mechanism can be simplified to the equivalent mechanism EDCF.

For four-bar mechanism EDCF, the cartesian coordinate system is established with node E as the origin and rod $l_{EF}$ as the X-axis, and the vector equation can be obtained:

(6) $$l_{ED}^{}+l_{DC}=l_{EF}+l_{FC}.$$

It is derived from Euler’s equations:

(7) $$\left\{ \begin{array}{l} l_{ED}\cos\alpha_{3}+l_{DC}\cos\alpha_{2}=l_{EF}\cos\alpha_{4}+l_{FC}\cos\alpha_{5}\\ l_{ED}\sin\alpha_{3}+l_{DC}\sin\alpha_{2}=l_{EF}\sin\alpha_{4}+l_{FC}\sin\alpha_{5} \end{array}\right..$$

In the equation, $\alpha_{4}=0$; (7) can be simplified as:

(8) $$\left\{ \begin{array}{l} l_{ED}\cos\alpha_{3}+l_{DC}\cos\alpha_{2}=l_{EF}+l_{FC}\cos\alpha_{5}\\ l_{ED}\sin\alpha_{3}+l_{DC}\sin\alpha_{2}=l_{FC}\sin\alpha_{5} \end{array}\right..$$

Subtracting $\alpha_{2}$ from (8) gives:

$$\alpha_{5}=\arcsin((bc\pm a\sqrt{a^{2}+b^{2}-c^{2}})/(a^{2}+b^{2}))$$

It should be taken in the EDCF four-bar mechanism:

$$\alpha_{5}=\arcsin((bc+a\sqrt{a^{2}+b^{2}-c^{2}})/(a^{2}+b^{2}))$$

The following can be obtained by substituting $\alpha_{5}$ into (8):

$$\alpha_{2}=\arctan((l_{FC}\sin\alpha_{5}-l_{ED}\sin\alpha_{3})/(l_{EF}+l_{FC}\cos\alpha_{5}-l_{ED}\cos\alpha_{3}))$$

In the equation above,

$$a=2(l_{FC}l_{EF}-l_{ED}l_{FC}\cos\alpha_{3}),b=2l_{ED}l_{FC}\sin\alpha_{3},\;c=l_{ED}^{2}-l_{DC}^{2}+l_{FC}^{2}+l_{EF}^{2}-2l_{ED}l_{EF}\cos\alpha_{3}.$$

#### 2.2.3. Kinematics Modeling in the Third Configuration

**Figure 6:**
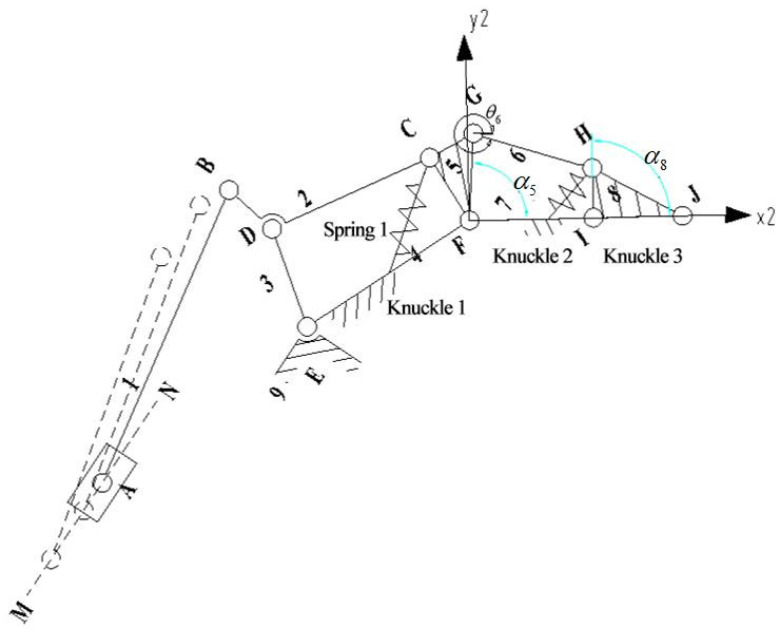
Schematic diagram of the equivalent mechanism of the third configuration.

When the second knuckle is in contact with the grasping object, it is the third configuration, as shown in Figure 6. At this point, the first and second knuckles remain static, and the third knuckle rotates around node I. The underactuated manipulator finger mechanism can be regarded as the linkage mechanism of two four-bar mechanisms, EDCF and FGHI. Note that the motion of rod 5 to rod 3 is the same as in the second configuration. 

For the four-bar mechanism FGHI, the cartesian coordinate system is established with node F as the origin and rod $l_{FI}$ as the X-axis, and the vector equation can be obtained:

(9) $${\vec{l}}_{FG}^{}+{\vec{l}}_{GH}={\vec{l}}_{FI}+{\vec{l}}_{IH}$$

It is derived from Euler’s equations:

(10) $$\left\{ \begin{array}{l} l_{FG}\cos\alpha_{5}+l_{GH}\cos\alpha_{6}=l_{FI}\cos\alpha_{7}+l_{IH}\cos\alpha_{8}\\ l_{FG}\sin\alpha_{5}+l_{GH}\sin\alpha_{6}=l_{FI}\sin\alpha_{7}+l_{IH}\sin\alpha_{8} \end{array}\right..$$

For convenience and calculation, the line between point F and point 1 indicates that rod 7 is placed horizontally, $\alpha_{7}=0$. Then, (10) can be simplified as:

(11) $$\left\{ \begin{array}{l} l_{FG}\cos\alpha_{5}+l_{GH}\cos\alpha_{6}=l_{FI}+l_{IH}\cos\alpha_{8}\\ l_{FG}\sin\alpha_{5}+l_{GH}\sin\alpha_{6}=l_{IH}\sin\alpha_{8} \end{array}\right..$$

Subtracting $\alpha_{6}$ from (11) gives:

$$\alpha_{8}=\arcsin((bc\pm a\sqrt{a^{2}+b^{2}-c^{2}})/(a^{2}+b^{2}))$$

It should be taken in the FGHI four-bar mechanism:

$$\alpha_{8}=\arcsin((bc+a\sqrt{a^{2}+b^{2}-c^{2}})/(a^{2}+b^{2}))$$

The following can be obtained by substituting $\alpha_{8}$ into (11):

$$\alpha_{6}=\arctan((l_{IH}\sin\alpha_{8}-l_{FG}\sin\alpha_{5})/(l_{FI}+l_{IH}\cos\alpha_{8}-l_{FG}\cos\alpha_{5}))$$

In the equation above,

$$a=2(l_{IH}l_{FI}-l_{FG}l_{IH}\cos\alpha_{5}),\;b=2l_{FG}l_{IH}\sin\alpha_{8},\;c=l_{FG}^{2}-l_{GH}^{2}+l_{IH}^{2}+l_{FI}^{2}-2l_{FG}l_{FI}\cos\alpha_{5}$$

### 2.3. Analysis of Contact Forces

To help the design and optimization of the underactuated finger, a static model of the finger was established, and its contact force was analyzed. To simplify the calculation process, the gravity of fingers and friction between fingers and objects were ignored.

**Figure 7:**
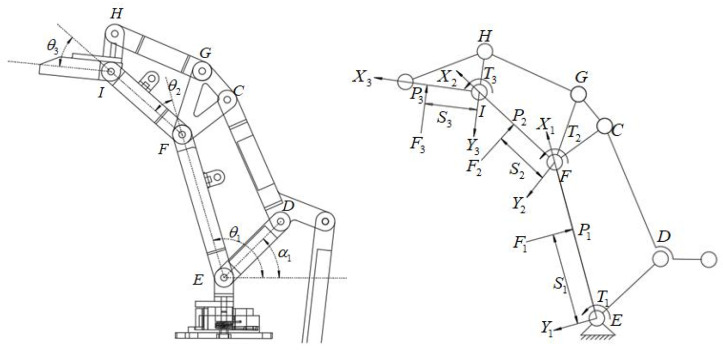
Geometric and static models of underactuated fingers.

The geometric and static mechanical models of the underactuated fingers are shown in Figure 7. When the fingers grasp the objects, the active rod drives the whole finger structure to move under the action of torque $T_{1}$. The three knuckles of the finger grab the object in order, and the contact points with the object are $P_{1}$, $P_{2}$, and $P_{3}$. The three knuckles are subjected to the reaction force $F_{1}$, $F_{2}$, and $F_{3}$ of the object. In addition, the torques produced by the elastic elements in the finger structure are $T_{2}$ and $T_{3}$; contact points $P_{1}$, $P_{2}$, and $P_{3}$ can be expressed as:

(12) $$P_{1}=(S_{1}\cos\theta_{1},S_{1}\sin\theta_{1}).$$

(13) $$P_{2}=(L_{1}\cos\theta_{1}+S_{2}\cos(\theta_{1}+\theta_{2}),L_{1}\sin\theta_{1}+S_{2}\sin(\theta_{1}+\theta_{2})).$$

(14) $$P_{3}=(L_{1}\cos\theta_{1}+L_{2}\cos(\theta_{1}+\theta_{2})+S_{3}\cos(\theta_{1}+\theta_{2}+\theta_{3}),L_{1}\sin\theta_{1}+L_{2}\sin(\theta_{1}+\theta_{2})+S_{3}\sin(\theta_{1}+\theta_{2}+\theta_{3})).$$

In the equation above, $L_{1}$, $L_{2}$, and $L_{3}$ are the lengths of the three knuckles; $\theta_{1}$, $\left(\theta_{1}+\theta_{2}\right)$ and $\left(\theta_{1}+\theta_{2}+\theta_{3}\right)$ are the included angles between the first, second, and third knuckles, respectively, and the horizontal direction; $S_{1}$, $S_{2}$, and $S_{3}$ are the distances between the contact points on the three knuckles and the joint axis.

The contact force between the object and the finger can be expressed as:

(15) $$\vec{F_{1}}=(\vec{F_{1}}\sin\theta_{1},-\vec{F_{1}}\cos\theta_{1})$$

(16) $$\vec{F_{2}}=(\vec{F_{2}}\sin\theta_{1},-\vec{F_{2}}\cos\theta_{1})$$

(17) $$\vec{F_{3}}=(\vec{F_{3}}\sin\theta_{1},-\vec{F_{3}}\cos\theta_{1})$$

According to the principle of virtual work:

(18) $$T^{T}\omega =F^{T}\nu$$

The following can be obtained according to (12)–(14):

(19) $$\overset{\cdot }{P_{1}}=(-S_{1}\overset{\cdot }{\theta_{1}}\sin\theta_{1},S_{1}\overset{\cdot }{\theta_{1}}\cos\theta_{1})$$

(20) $$\overset{\cdot }{P_{2}}=(-L_{1}\overset{\cdot }{\theta_{1}}\sin\theta_{1}-S_{2}\overset{\cdot }{\left(\theta_{1}+\theta_{2}\right)}\sin(\theta_{1}+\theta_{2}),L_{1}\overset{\cdot }{\theta_{1}}\cos\theta_{1}+S_{2}\overset{\cdot }{\left(\theta_{1}+\theta_{2}\right)}\cos(\theta_{1}+\theta_{2}))$$

$$\overset{\cdot }{P_{3}}=(-L_{1}\overset{\cdot }{\theta_{1}}\sin\theta_{1}-L_{2}\overset{\cdot }{\left(\theta_{1}+\theta_{2}\right)}\sin(\theta_{1}+\theta_{2})-S_{3}\overset{\cdot }{\left(\theta_{1}+\theta_{2}+\theta_{3}\right)}\sin(\theta_{1}+\theta_{2}+\theta_{3}),$$

(21) $$L_{1}\overset{\cdot }{\theta_{1}}\cos\theta_{1}+L_{2}\overset{\cdot }{\left(\theta_{1}+\theta_{2}\right)}\cos(\theta_{1}+\theta_{2})+S_{3}\overset{\cdot }{\left(\theta_{1}+\theta_{2}+\theta_{3}\right)}\cos(\theta_{1}+\theta_{2}+\theta_{3}))$$

By multiplying both ends of (19)–(21) by $F_{1}$, $F_{2}$, and $F_{3}$, respectively, we obtain:

(22) $$\vec{F_{1}}\cdot \overset{\cdot }{P_{1}}=-\vec{F_{1}}S_{1}\overset{\cdot }{\theta_{1}}$$

(23) $$\vec{F_{2}}\cdot \overset{\cdot }{P_{2}}=-\vec{F_{2}}(L_{1}\overset{\cdot }{\theta_{1}}\cos\theta_{2}+S_{2}\overset{\cdot }{\left(\theta_{1}+\theta_{2}\right)})$$

(24) $$\vec{F_{3}}\cdot \overset{\cdot }{P_{3}}=-\vec{F_{3}}(L_{1}\overset{\cdot }{\theta_{1}}\cos(\theta_{2}+\theta_{3})+L_{2}\overset{\cdot }{\theta_{2}}\cos\theta_{3}+S_{3}\overset{\cdot }{\left(\theta_{1}+\theta_{2}+\theta_{3}\right)})$$

Substituting (22)–(24) into (18) can obtain:

(25) $$\left[\begin{array}{ccc} \vec{F_{1}} & \vec{F_{2}} & \vec{F_{3}} \end{array}\right]\left[\begin{array}{c} \overset{\cdot }{P_{1}}\\ \overset{\cdot }{P_{2}}\\ \overset{\cdot }{P_{3}} \end{array}\right]=\left[\begin{array}{ccc} F_{1} & F_{2} & F_{3} \end{array}\right]\left[\begin{array}{ccc} J_{11} & 0 & 0\\ J_{21} & J_{22} & 0\\ J_{31} & J_{32} & J_{33} \end{array}\right]\left[\begin{array}{c} \overset{\cdot }{\theta_{1}}\\ \overset{\cdot }{\left(\theta_{1}+\theta_{2}\right)}\\ \overset{\cdot }{\left(\theta_{1}+\theta_{2}+\theta_{3}\right)} \end{array}\right]$$

$$J_{11}=-S_{1}$$

$$J_{21}=-L_{1}\cos\theta_{2}$$

$$J_{22}=-S_{2}$$

$$J_{31}=-L_{1}\cos(\theta_{2}+\theta_{3})$$

$$J_{32}=-L_{2}\cos\theta_{3}$$

$$J_{33}=-S_{3}$$

Combining (18)–(25), the contact force can be expressed as:

(26) $$F_{3}=\frac{-T_{3}}{S_{3}}=\frac{k\theta_{3}}{S_{3}}$$

(27) $$F_{2}=\frac{-T_{2}}{S_{2}}+\frac{T_{3}L_{2}\cos\theta_{3}}{S_{3}S_{2}}=\frac{k\theta_{2}}{S_{2}}-\frac{k\theta_{3}L_{2}\cos\theta_{3}}{S_{3}S_{2}}$$

$$F_{1}=\frac{-T_{1}}{S_{1}}+(\frac{T_{2}}{S_{2}}-\frac{T_{3}L_{2}\cos\theta_{3}}{S_{3}S_{2}})\frac{L_{1}\cos\theta_{2}}{S_{1}}+\frac{T_{3}L_{1}\cos(\theta_{2}+\theta_{3})}{S_{3}S_{1}}$$

(28) $$=\frac{-T_{1}}{S_{1}}-(\frac{k\theta_{2}}{S_{2}}-\frac{k\theta_{3}L_{2}\cos\theta_{3}}{S_{3}S_{2}})\frac{L_{1}\cos\theta_{2}}{S_{1}}-\frac{k\theta_{3}L_{1}\cos(\theta_{2}+\theta_{3})}{S_{3}S_{1}}$$

**Figure 8:**
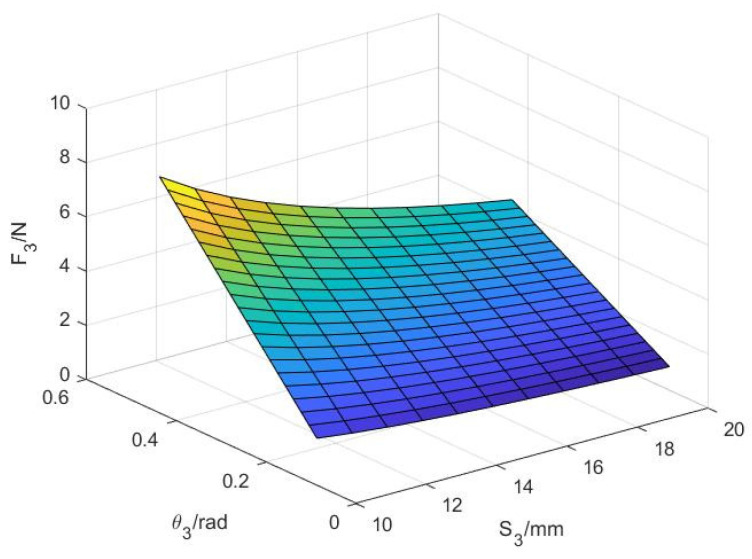
The functional relationship between variables $S_{3}$, $\theta_{3}$, and $F_{3}$.

According to the above, the relationship between the contact force, the contact point, and the rotation angle can be obtained. According to (26), the relationship between the contact force $F_{3}$ at the far finger and the angle $\theta_{3}$ and the distance $S_{3}$ of the contact point can be obtained during the grasping of the object by the envelope, as shown in Figure 8.

As shown in Figure 8, the contact force $F_{3}$ at the distal finger increases with the increase in the guide bar angle $\theta_{3}$. In addition, the smaller the position $S_{3}$ of the contact point on the distal finger, the greater the contact force on the distal finger. In other words, the closer the object is to the palm, the greater the contact force; this conforms to the situation of grasping objects by hand, indicating that the design is feasible.

**Figure 9:**
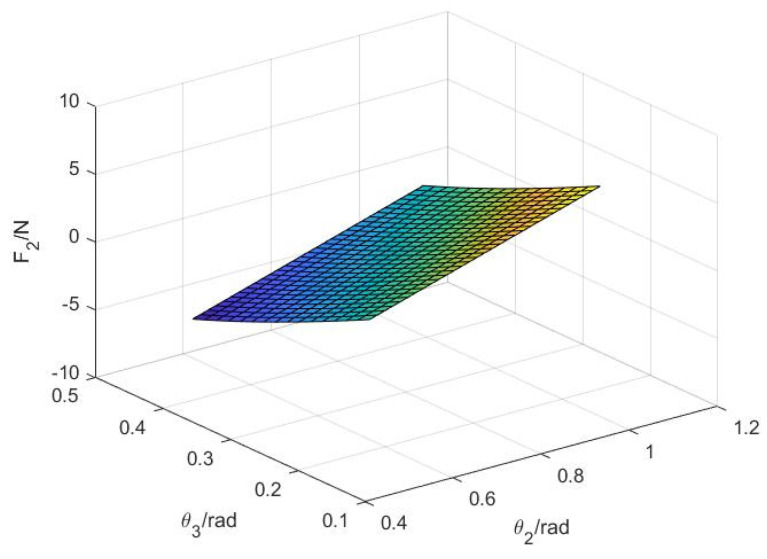
The functional relationship between variables $\theta_{2}$, $\theta_{3}$, and $F_{2}$.

Similarly, according to (27), the variation relationship between the second knuckle $F_{2}$, angle $\theta_{2}$, and angle $\theta_{3}$ can be obtained, as shown in Figure 9.

As shown in Figure 9, the contact force $F_{2}$ of the second knuckle increases with the increase of the angle $\theta_{2}$. When the angle $\theta_{2}$ is constant, $F_{2}$ decreases with an increase in angle $\theta_{3}$ because the third knuckle bears part of the contact force, which conforms to the situation of grasping objects by hands, indicating that the design is feasible.

**Figure 10:**
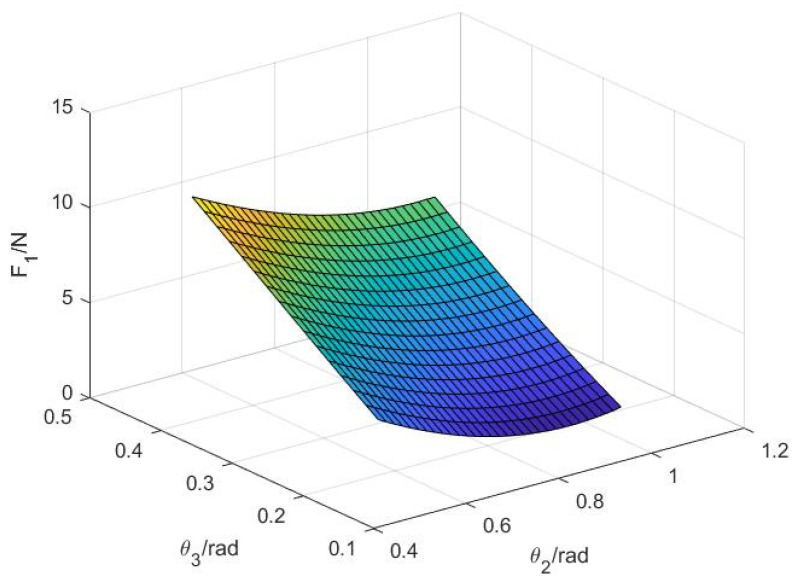
The functional relationship between variables $\theta_{2}$, $\theta_{3}$, and $F_{1}$.

Similarly, according to (28), the variation relationship between the second knuckle $F_{1}$, angle $\theta_{2}$, and angle $\theta_{3}$ can be obtained, as shown in Figure 10.

As can be seen in Figure 10, the contact force $F_{1}$ of the first knuckle decreases with an increase in angle $\theta_{2}$ because the second knuckle bears part of the contact force. However, when the angle $\theta_{2}$ is certain, $F_{1}$ increases with an increase in the angle $\theta_{3}$. This is because when the third knuckle begins to bear the contact force, the envelope of the first knuckle is tighter to grasp the object, which conforms to the situation of grasping the object by hand, indicating that the design is feasible.

### 2.4. Optimization of Finger Structure Parameters

The purpose of the underactuated finger mechanism is to achieve a reliable grasping function, which requires enough grasping force under the action of the limited driving force; that is, enough contact force can be generated between the finger and the object, and the force not so large that it causes damage to the grasping object. For grasping the finger mechanism in a stable state, the basic requirements are that the contact force of finger knuckles is as uniform as possible, the finger mechanism is compact in design, and good force transmission characteristics.

According to (26)–(28), the calculation models of the three knuckle contact forces have two characteristics: multiple variables and multiple parameters. The calculation of contact forces is very complicated, and it is difficult to identify the influence of each parameter on the contact force, which makes it a key problem to determine the design parameters of finger mechanisms [20].

#### 2.4.1. Determination of Finger Structural Parameters

From the perspective of bionics, the size of objects that human hands can grasp is closely related to the length of the fingers. Therefore, the length of each finger can be determined by the size of the object to grab. Taking an apple as an example and referring to the structure size of a human finger, the length of finger knuckles is determined as $L_{1}=60\;\mathrm{mm}$, $L_{2}=40\;\mathrm{mm}$, $L_{3}=32\;\mathrm{mm}$. After determining the basic length of each knuckle of the finger mechanism, other structural parameters of the finger mechanism need to be determined.

**Figure 11:**
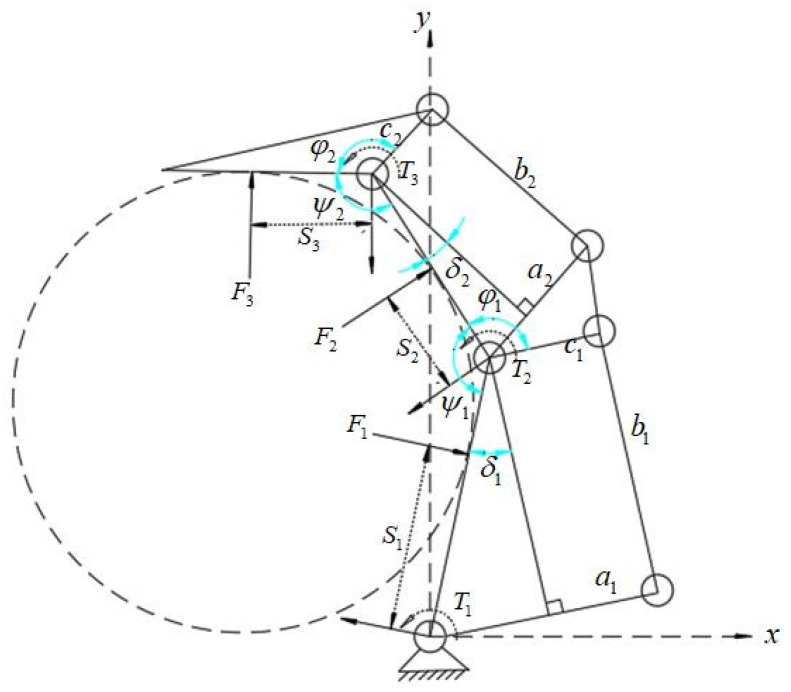
Schematic diagram of ideal grasping state.

According to the mechanical principle, when the transmission pressure angle of the mechanism is zero, the force transmission efficiency is the highest. Therefore, if $a_{1}$ is perpendicular to $b_{1}$, $c_{1}$ is perpendicular to $b_{1}$, $a_{2}$ is perpendicular to $b_{2}$, and $c_{2}$ is perpendicular to $b_{2}$, the finger mechanism can ensure the best force transmission effect, and thus the grasping state of the finger mechanism, as shown in Figure 11, is obtained. To ensure that the finger mechanism has a high force transmission effect in the whole grasping range, this state is set at the middle value of the relative angle of the knuckle, namely $\psi_{1}=\psi_{2}=135^{{}^{\circ}}$.

#### 2.4.2. Optimization of Objective Functions and Design Variables

According to the design requirement that the finger mechanism should try to achieve the uniform distribution of the contact force of each knuckle [17], let:

$$f_{1}=\max(F_{1},F_{2},F_{3})$$

$$f_{2}=\min(F_{1},F_{2},F_{3})$$

Then, the objective function of mechanism parameter optimization is:

(29) $$f=abs(f_{1}-f_{2})$$

Here are the geometric constraints:

(30) $$\{\begin{array}{c} \delta_{1}=\arcsin\frac{a_{1}-c_{1}}{L_{1}}\\ \delta_{2}=\arcsin\frac{a_{2}-c_{2}}{L_{2}} \end{array}$$

(31) $$\{\begin{array}{c} \varphi_{1}=1.5\pi -\delta_{1}-\psi_{1}\\ \varphi_{2}=1.5\pi -\delta_{2}-\psi_{2} \end{array}$$

(32) $$\{\begin{array}{c} T_{2}=-\left(k\times \pi /4+\tau \right)\\ T_{3}=-\left(k\times 5\pi /36+\tau \right) \end{array}$$

(33) $$\{\begin{array}{c} b_{1}=\sqrt{L_{1}^{2}-{\left(a_{1}-c_{1}\right)}^{2}}\\ b_{2}=\sqrt{L_{2}^{2}-{\left(a_{2}-c_{2}\right)}^{2}} \end{array}$$

The known parameters are $L_{1}=60\;\mathrm{mm}$, $L_{2}=40\;\mathrm{mm}$, $L_{3}=32\;\mathrm{mm}$. Due to the specific position of the finger mechanism, $T_{2}$ and $T_{3}$ are determined by the torsion spring stiffness k and the initial torque τ. The angle parameters $\varphi_{1}$ and $\varphi_{2}$ as well as the connecting rod length parameters $b_{1}$ and $b_{2}$ can be expressed by the finger knuckle length parameters $a_{1}$, $a_{2}$, $c_{1}$, and $c_{2}$.

According to the contact force expression in the objective function (26)–(28), $a_{1}$, $a_{2}$, $c_{1}$, $c_{2}$, k, τ were taken as the design variables of the finger mechanism. Genetic algorithm tools in MATLAB were used for analysis and calculation. The constraint range of ($a_{1}$, $a_{2}$, $c_{1}$, $c_{2}$, k, τ) was (30, 25, 20, 16, 3, 50); (45, 40, 35, 25, 10, 300), the population number was selected as 5000, the stasis selection algebra was set at 50, and the weighted average change of fitness function was less than 10^−6^. When any of the above conditions were met, the algorithm stopped. The algorithm was run independently several times, and the following 10 groups of data were selected to obtain the design parameters shown in Table 1.

The fourth group of data was selected as the design parameter. Rounding the parameters, $a_{1}=45\;\mathrm{mm},a_{2}=38\;\mathrm{mm},c_{1}=18\;\mathrm{mm},c_{2}=18\;\mathrm{mm}$, $k=9.4\;N\cdot \mathrm{mm}{(}^{0})$, τ=77 N·mm. Assuming the driving torque, $T_{1}=1000\;N\cdot \mathrm{mm}$, $F_{1}=12.59\;N$, $F_{2}=11.41\;N$, $F_{3}=11.56\;N$ could be obtained through calculation. This conformed to the conditions of uniform stress and met the requirements of the design, therefore, it could be used as the basis of structural design.

## 3. Experiments and Results

On the basis of the mechanism design, mechanical analysis, and structural parameter optimization of the underactuated manipulator, the corresponding control system was designed, and the prototype of the manipulator was made. The rationality of the underactuated hand structure was verified by grasping different objects, and further study of the underactuated hand was prepared.

### 3.1. Control System Composition

**Figure 12:**
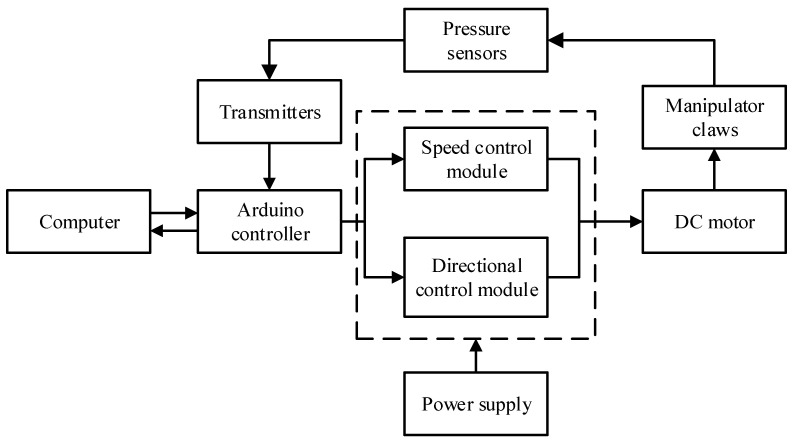
Schematic diagram of the control system.

The hardware of the manipulator control system was mainly composed of an Arduino control board, DC motor and supporting speed controller, direction controller, pressure sensors, AC–DC voltage converter, host computer, etc. The control system diagram is shown in Figure 12.

The speed control signal and direction control signal were sent through the Arduino control board to drive the DC motor to work; the pressure and speed were controlled by the converted voltage signal feedback from the film pressure sensor; and the pressure data could be displayed on the host computer in real-time.

**Figure 13:**
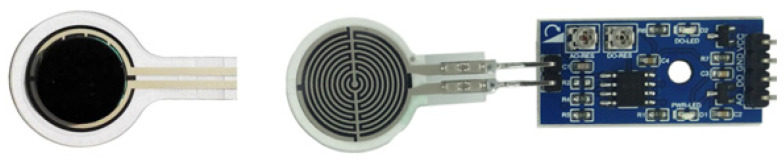
Thin-film pressure sensors and transmitters.

The pressure sensor adopted a resistive film pressure sensor, as shown in Figure 13; Table 2 is resistive film pressure sensor parameter: the range of the sensor was 0~10 kg, the film thickness was less than 0.3 mm, and the response time was less than 1 ms. The specific parameters are shown in Table 1. The sensor was pasted on the center of the mechanical finger, and the film sheet was pasted with a rubber pad, which facilitated better contact with the surface of the object and measured the contact pressure. The sensor was connected to the pressure transmitter; the transmitter could convert the pressure data into an analog voltage signal output, and the voltage signal could be easily read by the analog input interface of the Arduino control board and processed by the computer.

### 3.2. Closed-Loop Control Grasping Experiment

**Figure 14:**
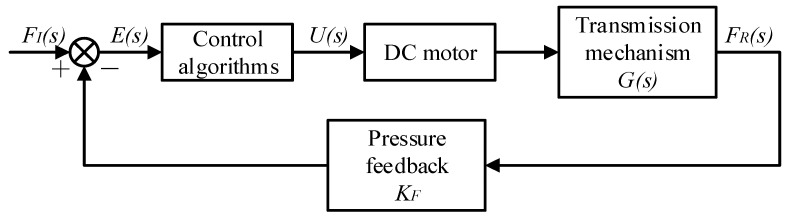
Control system structure drawing.

To maintain the stable grasping of the finger joints, it was necessary to carry out closed-loop control of the driving system of the motor (Figure 14). The motor speed was controlled by the principle of pulse width modulation (PWM), that is, a target pressure value was set through the computer. When the manipulator was not in contact with the object, the measured pressure value was 0, and the pressure difference was large. A large duty cycle of the motor was set, and the motor rotated quickly to achieve a fast grasping function. When it started to touch the object and drive the manipulator to metamorphose, the measured pressure value increased continuously, and the difference from the set value gradually decreased. Given a small duty cycle, the motor rotated slowly to achieve a stable grasping function; when the pressure setpoint was reached, the motor stopped. If the motor was not stopped due to the fluctuation of the measured pressure, the manipulator continued to apply force to the object at this time, and the measured pressure value was greater than the set value. By setting the computer program to determine that the difference was less than 0, the duty cycle was quickly reduced so that the motor decelerated and stopped. In this way, the force could be roughly maintained near the set value, so the control of the force was reflected in the control of the motor.

The underactuated manipulator designed in this study had only one motor drive input but three pressure outputs, and it was impossible to control all of them. Through experiments on different joints of one finger and the same joint of different fingers, the experimental results were compared.

### 3.3. Analysis of the Effect of Grasping Experiment

**Figure 15:**
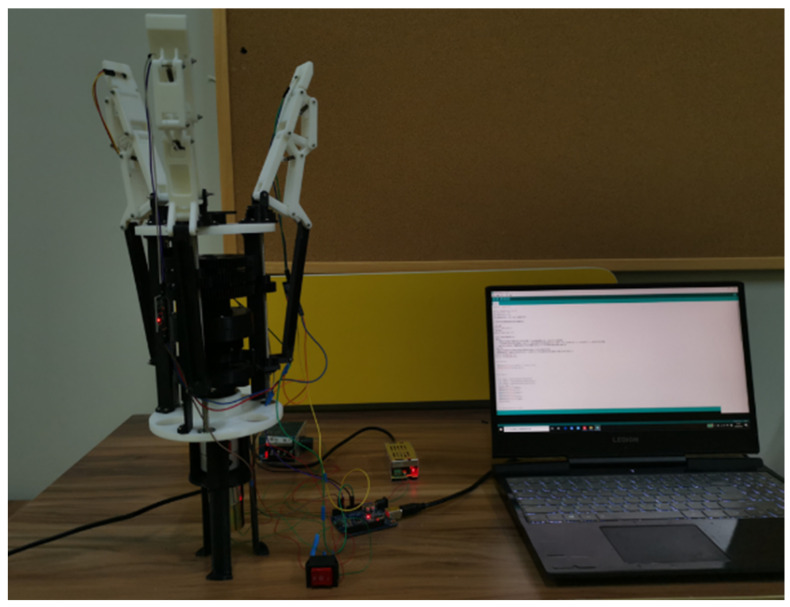
Underactuated manipulator.

Figure 15 shows the experimental prototype of the underactuated manipulator designed in this study based on the metamorphic principle. The grasping experiment of the underactuated hand was mainly conducted to observe the underactuated hand grasping target objects within a certain volume and mass range. To better verify the grasping ability of underactuated hands, cylindrical objects and spherical objects commonly used in daily life were mainly selected. The power of the dc motor was 10 W, the diameter of the bottom of the cylindrical object was 70 mm, and the length was 130 mm; the diameter of the spherical object was 100 mm.

**Figure 16:**
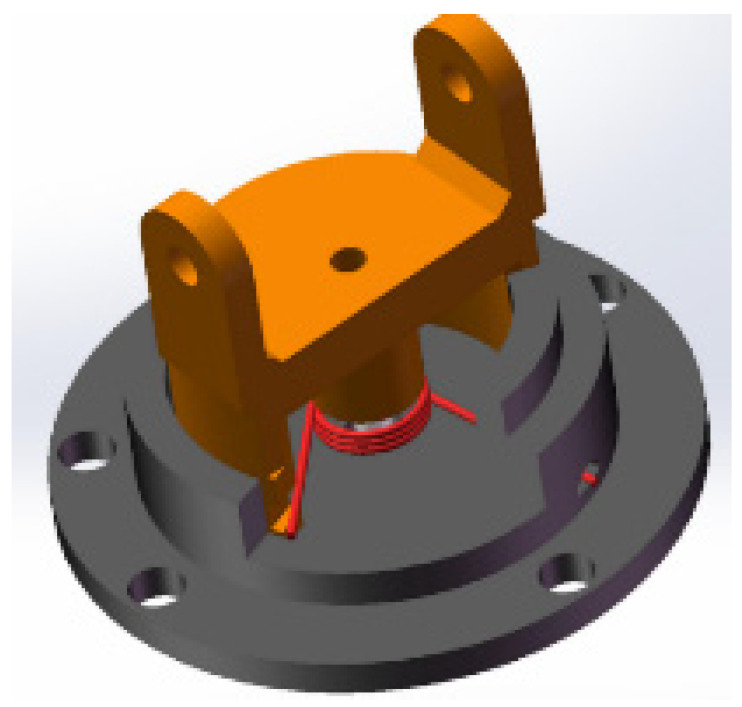
Finger branch adaptive structure.

**Figure 17:**
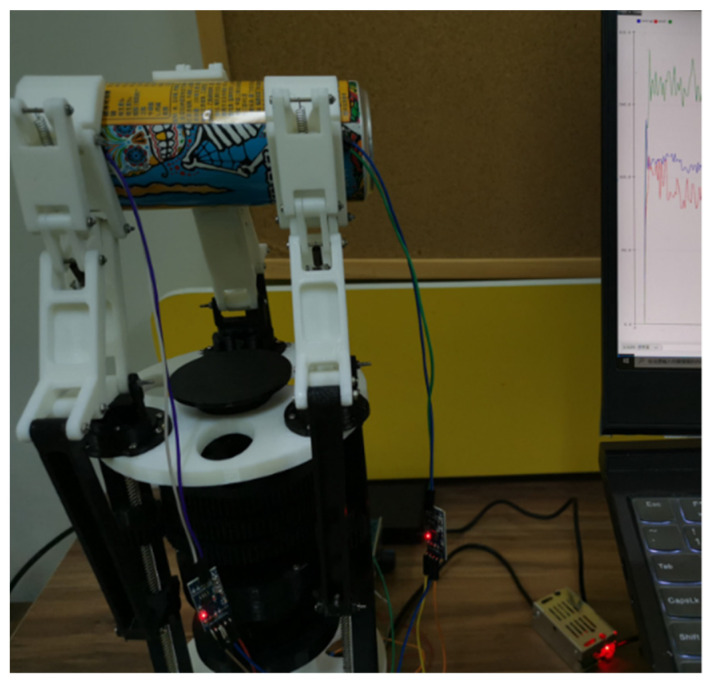
Grabbing a cylinder experiment.

**Figure 18:**
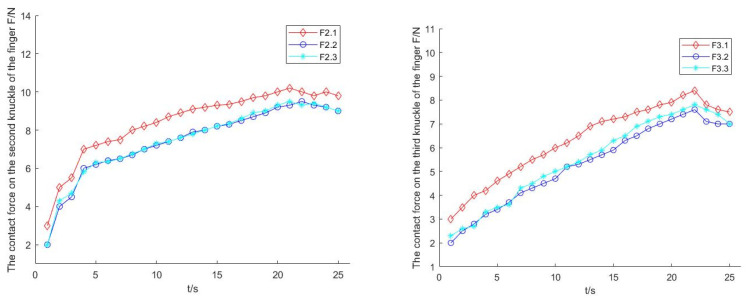
Contact force of the second and third knuckles when grasping the cylinder.

In the process of enveloping and grasping cylindrical objects, due to the adaptive structure of finger branches at the base of the fingers, as shown in Figure 16 and Figure 17, fingers 2 and 3 could adjust the pose for grasping, and the contact force between the two when grasping the object was equal or close in size. In addition, it can be seen in Figure 18 that due to the balance of forces, the force on finger 1 was larger than that on finger 2 and finger 3.

**Figure 19:**
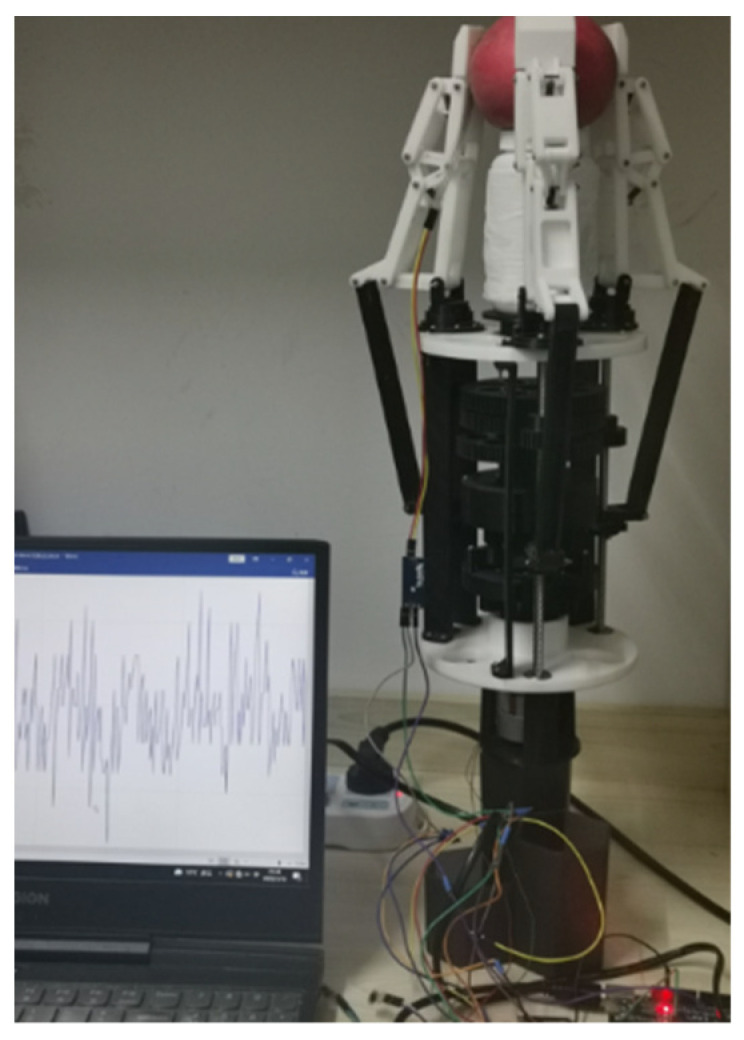
Grabbing an apple experiment.

**Figure 20:**
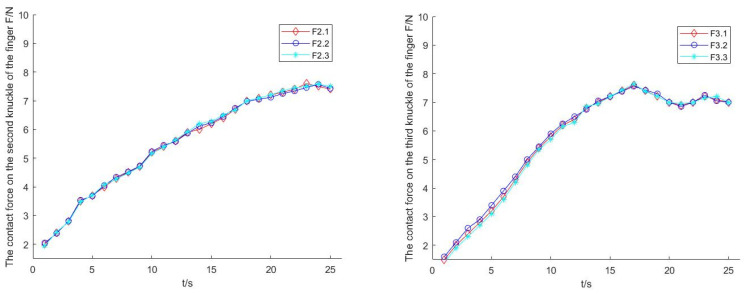
Contact force of the second and third knuckles when grasping the ball.

When the manipulator grabbed a spherical object by envelope, as shown in Figure 19, such as an apple, the contact force between the second and third knuckles of the three fingers and the apple was similar, and the contact force is shown in Figure 20.

Through the prototype experiment of an underactuated hand, it was shown that the manipulator has the following advantages:

1. Underdrive: Each finger has three degrees of freedom, and one motor is enough to drive. This means the fingers are easy to control and adaptable.
2. Ability to switch configuration during grasping: Each finger of the underactuated manipulator carries out configuration transformation in the process of grasping, and they cannot interfere with each other.
3. Self-adaptability and envelope grabbing ability: By grasping different objects, the second and third knuckles of the underactuated manipulator can grasp objects of different sizes and positions stably.
4. Strong grasp ability: According to the current drive motor power, in the case of limited drive, the capacity of the grabbing load was 20 N. Through the grasping the cylinder experiment, in the process of grasping, the force exerted by the second finger was the largest, and the maximum value was close to 10 N, indicating that the manipulator has a strong ability to obtain external objects.

## 4. Conclusions

On the basis of the metamorphic mechanism, an underactuated robot hand was designed in this study. The structure design, kinematics, contact force, and optimization of finger structure parameters were discussed, and a prototype was made for the grasping test.

Kinematic analysis was used to analyze the different configuration transformations of finger mechanisms in the grasping process. The contact force analysis revealed the contact force of each knuckle when grasping different objects, which provides a theoretical reference for the design and manufacturing process.

Usually, the size of the object determines the length of the finger knuckle. When the basic length of each knuckle is determined, other structural parameters of the finger mechanism should be optimized to achieve enough grasping force under the action of the limited driving force. The contact force of finger knuckles should be as uniform as possible, and the finger mechanism should be compact and have good force transmission characteristics.

Through grasping experiments, it was verified that the manipulator could carry out configuration changes and grasp objects of different sizes, positions, and shapes.

## Figures and Tables

**Table 1:** Structural parameters of each knuckle of the finger.

	Length of Each Knuckle/mm				Spring Stiffness	Initial Torque	The Objective Function
	$a_{1}$	$a_{2}$	$c_{1}$	$c_{2}$	$k/[N\cdot \mathrm{mm}/{(}^{0})]$	τ/(N·mm)	$f/\left(1\times 10^{-7}N\right)$
1	42.5	38.4	26.0	21.9	4.41	72.2	18.0
2	38.6	31.1	23.5	17.4	6.34	70.3	17.1
3	44.6	25.5	22.8	19.7	9.36	50.8	9.40
4	44.9	38.4	24.9	18.2	9.42	77.1	5.79
5	41.9	31.8	21.3	18.7	5.99	59.4	12.2
6	38.4	37.4	21.8	19.5	4.48	54.7	14.8
7	42.4	27.8	20.6	23.2	9.30	57.8	11.2
8	44.8	29.7	21.0	17.8	5.40	51.6	16.2
9	44.5	30.8	21.3	18.4	8.46	54.5	8.90
10	42.2	28.8	20.5	22.6	7.62	109	3.76

**Table 2:** Resistive film pressure sensor parameter table.

Type	DF9-40
Range	0~10 kg
Thickness	≤0.3 mm
Repeatability	±5% (50% load)
Durability	<1 million times
Response time	>1 ms
Recovery Time	>15 ms
Operating temperature	−20 °C~60 °C
